# Bacterial resistance to CRISPR-Cas antimicrobials

**DOI:** 10.1038/s41598-021-96735-4

**Published:** 2021-08-26

**Authors:** Ruben V. Uribe, Christin Rathmer, Leonie Johanna Jahn, Mostafa Mostafa Hashim Ellabaan, Simone S. Li, Morten Otto Alexander Sommer

**Affiliations:** 1 Novo Nordisk Foundation Center for Biosustainability, Technical University of Denmark, Kgs. Lyngby, Denmark; 2 School of Chemistry and Molecular Biosciences, University of Queensland, Brisbane, QLD Australia

**Keywords:** Biotechnology, Microbiology, Molecular biology

## Abstract

In the age of antibiotic resistance and precise microbiome engineering, CRISPR-Cas antimicrobials promise to have a substantial impact on the way we treat diseases in the future. However, the efficacy of these antimicrobials and their mechanisms of resistance remain to be elucidated. We systematically investigated how a target *E. coli* strain can escape killing by episomally-encoded CRISPR-Cas9 antimicrobials. Using Cas9 from *Streptococcus pyogenes* (SpCas9) we studied the killing efficiency and resistance mutation rate towards CRISPR-Cas9 antimicrobials and elucidated the underlying genetic alterations. We find that killing efficiency is not correlated with the number of cutting sites or the type of target. While the number of targets did not significantly affect efficiency of killing, it did reduce the emergence of chromosomal mutations conferring resistance. The most frequent target of resistance mutations was the plasmid-encoded SpCas9 that was inactivated by bacterial genome rearrangements involving translocation of mobile genetic elements such as insertion elements. This resistance mechanism can be overcome by re-introduction of an intact copy of SpCas9. The work presented here provides a guide to design strategies that reduce resistance and improve the activity of CRISPR-Cas antimicrobials.

## Introduction

Antibiotic resistance is undermining the achievements of modern medicine as infectious diseases are becoming increasingly threatening again due to the global rise of antibiotic resistant pathogens^1^. The crisis is further enhanced by a lack of new therapeutics. The development of novel antibiotics is a cost and time intensive venture and only few companies invest in antibiotic drug discovery today^2^. New treatment strategies against antibiotic resistant bacteria are urgently needed in order to combat resistant bacteria.

A fast development in the field of synthetic biology based on CRISPR (clustered regularly interspaced short palindromic repeats) systems might revolutionize the way we treat disease in the future. The promising technology stems from the adaptive immune systems from bacteria and archaea^3^. Their Cas (CRISPR-associated) nucleases recognize a specific sequence of DNA by forming a complex with a CRISPR-RNA (crRNA) that has sequence homology to the target^4,5^. The crRNA-Cas complex binds to the target and introduces a DNA break. Due to the precision of the CRISPR-Cas system and ease of programmability, CRISPR-based tools for genome editing have been successfully applied in eukaryotes and prokaryotes, where damaged DNA is repaired via homologous recombination (HR) using a matching copy of DNA^6–8^. Alternatively, eukaryotic cells can repair DNA breaks using the error-prone non-homologues end joining (NHEJ) mechanisms^9^. However, most prokaryotes lack NHEJ mechanisms, wherefore continuous DNA damage leads to cell death if not repaired through homologous recombination^10^. This phenomenon has been exploited for the development of CRISPR-Cas based antimicrobials^11–13^.

CRISPR-Cas antimicrobials have the advantage over antibiotics to discriminate and eliminate specific bacteria at the strain level. Antibiotic treatment is often associated with a change in the human microbiome leading to a temporary reduction of diversity, which might increase the risk for other diseases like *Clostridium difficile* infection^14–16^. Therefore, a drug that specifically targets pathogenic bacteria, while leaving the rest of the microbiota intact, would be highly beneficial. Moreover, resistant bacteria could be targeted specifically, clearing them from the infection and leaving antibiotic susceptible bacteria that can be eliminated by standard antibiotic treatments^17^. In addition, the technique could also be used for targeted microbiome engineering, highlighting the potential of CRISPR-Cas based medicine to not only treat infectious diseases but also multiple microbiome-related conditions such as diabetes^18^, obesity^19^ and cancer^20,21^.

The basic design of CRISPR-Cas antimicrobials consists of a crRNA encoded in a DNA vector and an endogenously or exogenously provided CRISPR-Cas effector. These systems are remarkably diverse in both the structural components and functions. Their different effector modules can target DNA, RNA or both^22^. The most commonly studied effector protein is the DNA endonuclease Cas9 from *Streptococcus pyogenes* (SpCas9) using a specificity-programming guideRNA (gRNA)^4,23^. In previous reports, SpCas9 has been reprogrammed and successfully deployed to induce bacterial cell death in antibiotic resistant and clinically relevant bacteria^11,12,24,25^.

Yet, one of the main challenges of this technology is the relatively high rate of bacteria escaping CRISPR-Cas double stranded DNA break potentially due to mutations in CRISPR-Cas effector protein, gRNA or the target sequence^11,12,26–28^. Furthermore, in many cases escaper bacteria are the result of intrinsic resistance or tolerance to CRISPR-Cas antimicrobials that is associated with gRNA efficiency and the activity of RecA-mediated DNA repair^29–31^. After DNA damage, the ubiquitous RecA system repairs the break using an intact chromosomal copy of the cleaved DNA strand^29^, therefore if a weak target is chosen, the rate of RecA-mediated DNA repair will be higher than SpCas9 induced double stranded breaks resulting in a higher survival rate.

As intrinsic and acquired resistance is frequently observed as an immediate response to in vitro treatment of bacteria with CRISPR-Cas based antimicrobials, it is crucial to understand the underlying mechanisms in order to improve the therapeutic efficiency. We set out to study some of the parameters affecting the CRISPR-Cas killing efficiency and resistance rates.

## Results

### Multiple cutting site does not improve killing efficiency

**Figure 1:**
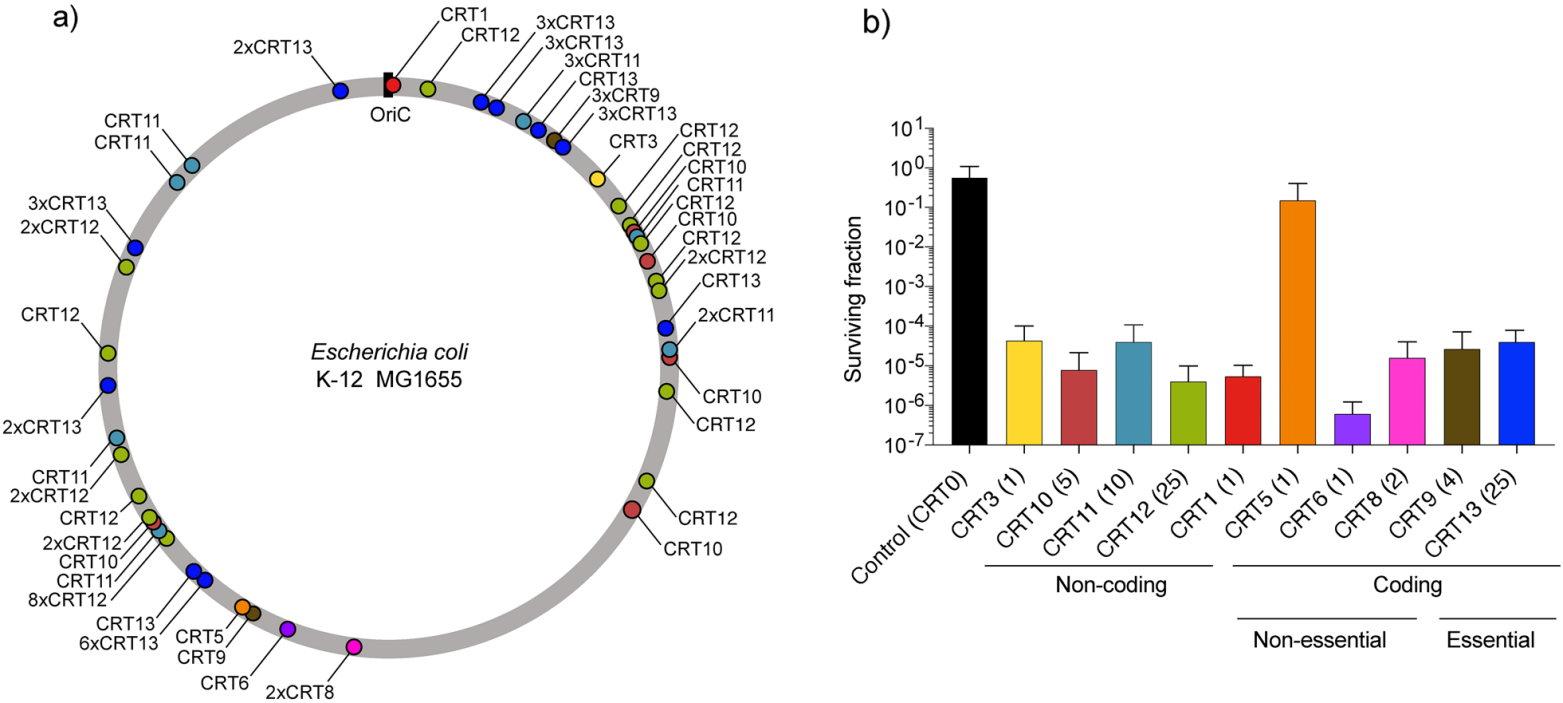
gRNA targets in the chromosome of *E. coli* K-12 MG1655. (**a**) Chromosomal map of *E. coli* K-12 MG1655 indicating the oriC and the cutting sites of the different CRISPR targets (CRTs). (**b**) Efficiency of the CRTs determined by the surviving fraction, the CRTs are ordered by gRNAs targeting non-coding vs. coding regions, non-essential vs. essential genes and the number of cutting sites. CRT0 indicated as a black bar served as a control gRNA with a non-targeting sequence.

Tolerance against double stranded-DNA breaks caused by CRISPR-Cas is often associated with specific sites in the chromosome^29–31^. Therefore, it has been suggested that the number of target sites could reduce the number of cells escaping double stranded break and improve the killing efficiency of CRISPR-Cas based antimicrobials^13,32^. To systematically analyze the impact of multiple chromosomal double stranded breaks, 10 different gRNAs were selected with between 1 and 25 sites in the genome of *E. coli* MG1655 K12 (EC0000096) (Fig. 1a and Supp. Table s2). Additionally, as a control we included a gRNA with no target (CRT0). The different gRNAs pair with the type II effector protein SpCas9 to introduce double stranded breaks in one or multiple sites in the chromosome. The expression of SpCas9 was coupled to a theophylline riboswitch, while the gRNA was under an arabinose inducible promoter. The killing efficiency was determined after induction of gRNA and SpCas9 compared to an uninduced control.

In contrast to our hypothesis that the number of target sites would be linked to the killing efficiency, no correlation was observed (Fig. 1b). Overall the killing efficiency was around 99.99% of the total population of bacteria expressing SpCas9 and the corresponding gRNA. Particularly, CRT5 had the lowest killing efficiency (84.9%), which is consistent with the predicted low activity score^31^ (Supp. Table s2). Interestingly, CRT6, had also only one target site but its performance was 99.999% with an efficiency among the highest (Fig. 1b).

The distance to the origin of replication (oriC) as well as coding versus non-coding regions influence the accessibility of the DNA^33^. The target sites were chosen in a manner that they were randomly spread around the whole genome and located in both coding and noncoding regions (Fig. 1a and Supp. Table s2). However, the target site distance from the origin did not seem to impact killing efficiencies. Furthermore, the number of cutting sites does not appear to influence the scale of the efficiency. Neither did it impact killing efficiency if a coding or a non-coding region was targeted in the genome (Fig. 1b).

The gRNA design rules underlying strong activity are not well understood; however, several parameters are believed to influence the activity of the gRNA:SpCas9 in the genome, including supercoiling of DNA and torsional constraints^34^. Libraries of thousands of gRNAs have been tested to facilitate scoring of the gRNA activity across the genome of *E. coli*^30,31^. These data were utilized to build a model for predicting gRNA activity. We compared our measured efficiency with the predicted ranking based on a model by Guo et al. (2018). While some results are overlapping such as a low activity for CRT5 and CRT13, no clear correlation could be obtained (Fig. 1b and Supp. Figure s1). The differences between the predicted activity scores and our results could be attributed to the differences in the experimental setup, for instance the expression level of SpCas9 or the fact the model from Guo et al. (2018) does not account for targets with multiple cutting sites.

### CRISPR-SpCas9 expression impacts killing efficiency

**Figure 2:**
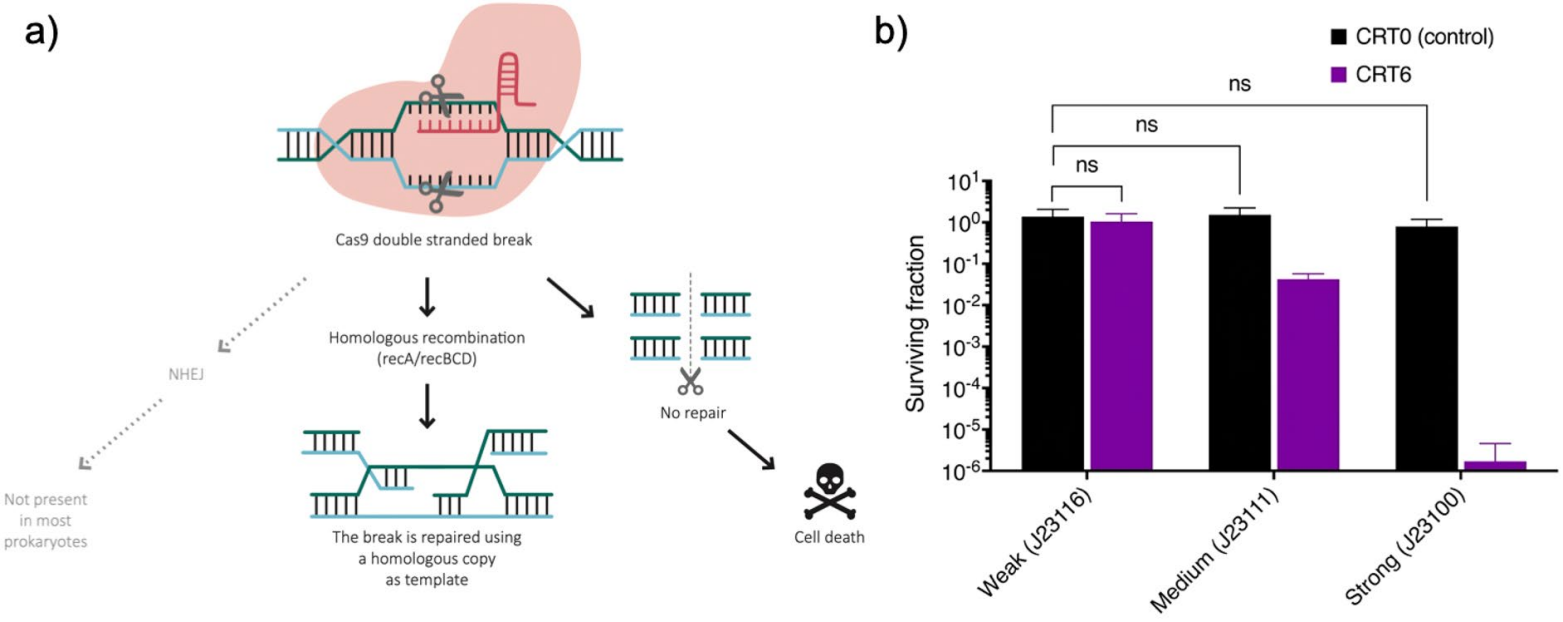
Mechanism of CRISPR-Cas antimicrobials and killing efficiency of different expression levels of SpCas9. (**a**) SpCas9-double stranded break results in DNA damage. In order to repair it, most prokaryotes rely almost exclusively on RecA-mediated HR using an intact copy as substitute DNA template, however if the DNA damage is not repaired the consequences are lethal for the cell. In the context of CRISPR-Cas antimicrobials, cell death is the outcome when the DNA damage is faster than the repair mechanisms of the cell. (**b**) The fraction of bacteria surviving SpCas9-mediated death was determined by plating the same dilution of cells on agar plates with and without the inducers (2 mM of theophylline and 1% arabinose). CRT0 (control) is a guide RNA that has no target site in the chromosome of *E. coli* MG1655. CRT6 is a gRNA with a single target site. SpCas9 was expressed from 3 promoters with distinct expression strength based on the scores from the Anderson collection (http://parts.igem.org/Promoters/Catalog/Anderson): weak (J23116), medium (J23111) and strong (J23100) promoters.

When SpCas9-mediated double stranded break occurs, most bacteria repair it using the ubiquitous RecA-mediated pathway. Since this is the main mechanism used in prokaryotes, failure in repairing the DNA has lethal consequences. The role of RecA mediating the efficiency of SpCas9 has been investigated elsewhere^29–31^, instead we investigated the role of SpCas9 expression level for the intrinsic resistance or tolerance associated to CRISPR-Cas antimicrobials. The level of SpCas9 expression is one parameter often overlooked in various studies. For instance, when SpCas9 is mildly expressed bacteria can tolerate an active self-targeting gRNA, without up-regulating DNA repair genes^35^. We hypothesize that *E. coli* “escapers” can survive chromosomal double stranded breaks when the level of active SpCas9 is too low to overcome the RecA-mediated repair (Fig. 2a). Therefore, we designed three genetic circuits based on different promoter strengths previously characterized^36^. We choose the promoters J23116, J23111 and J23100 for weak, intermediate and strong expression of SpCas9, respectively. The killing efficiency was evaluated using CRT6, which is a potent single-target gRNA and CRT0 as a control.

The fraction of surviving bacteria was determined after induction of gRNA and SpCas9 compared to the uninduced control. The expression strength was clearly linked to the killing efficiency of the system (Fig. 2b). Although the CRT6-target site had a high efficiency before, changing the level of SpCas9 expression in the cells resulted in more cells escaping the double stranded break. These results indicate that even a highly efficient gRNA targeting the chromosome is not enough to cause lethal effects in the cells. Instead we demonstrate that the level of SpCas9 is an important parameter to take into account in order to reduce the number of cells surviving double stranded breaks.

### SpCas9 effector is the preferred target for mutations

**Figure 3:**
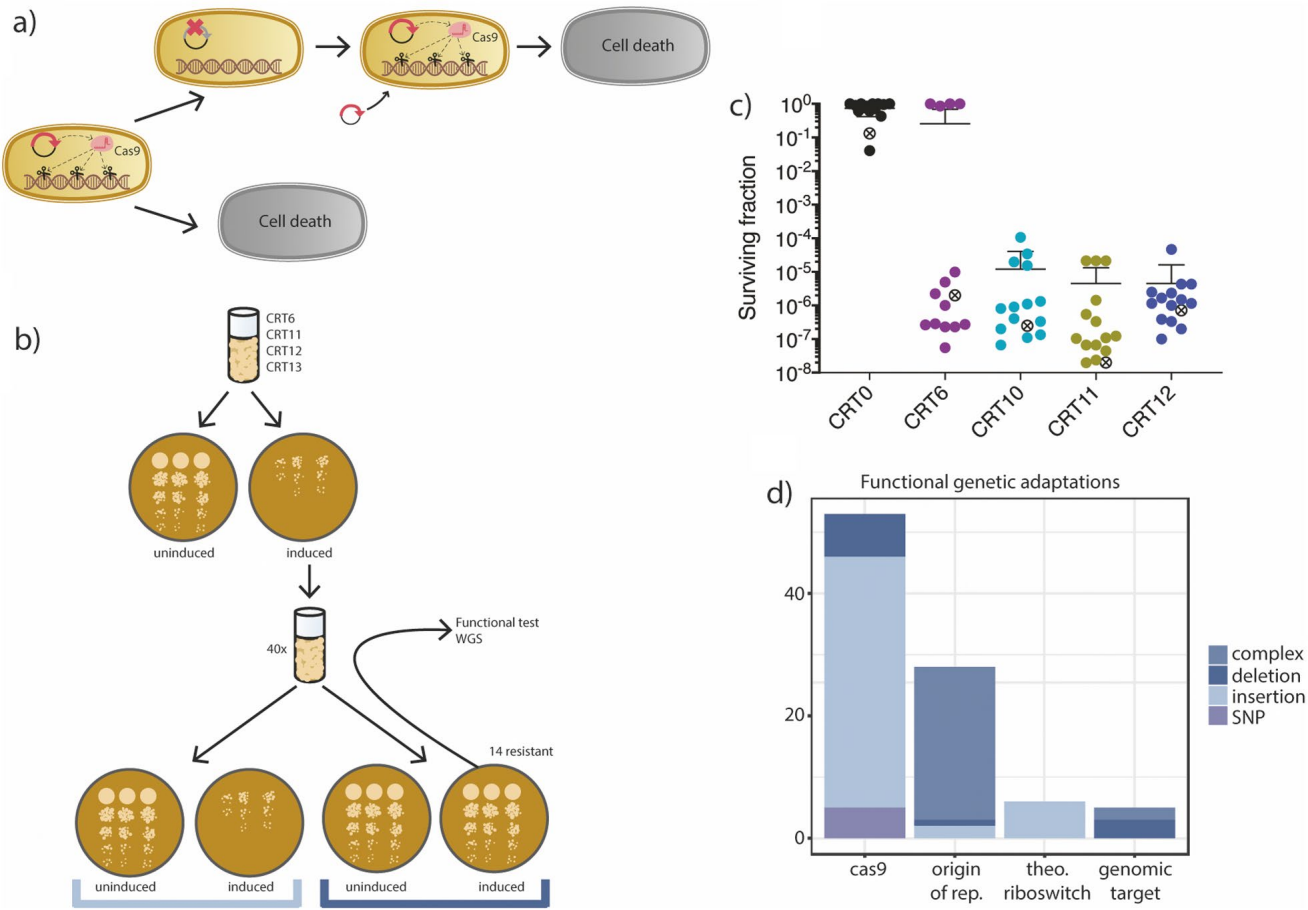
Reintroduction of CRISPR-Cas antimicrobial. (**a**) Schematic description of plasmid re-introduction. After SpCas9 induced death, escaper cells have inactivated the antimicrobial by mutating *cas9*. In order to re-sensitize the cells, a new SpCas9 plasmid is delivered to the escape mutants. (**b**) Workflow for re-introduction of CRISPR-Cas antimicrobials and selection of mutants for whole genome sequencing analysis (WGS). (**c**) Surviving fraction of 14 mutants after reintroduction of an intact spCas9 copy. (**d**) Frequency of complex mutations, deletions, insertions and single nucleotide polymorphisms (SNPs) on the *cas9* carrying plasmid pCasens3 and at the genomic target sites of CRT0 (fucI) and CRT6 (fadD) identified for each sequenced lineage. Complex mutations include a combination of multiple structural variants or inversions, as defined by CLC Genomic Workbench. 14 biological replicates were included of CRT0, CRT6, CRT11, CRT12 and CRT13 as well as a uninduced control.

Further we were interested in investigating whether the bacteria that survived SpCas9 mediated double stranded breaks were tolerating the stress or if they acquired mutations that prevented killing. Thus, in order to distinguish bacteria tolerating double stranded break from the ones that acquired beneficial mutations, we subjected 40 escaper colonies of each experiment from: CRT0 (control), CRT6, CRT11, CRT12 and CRT13 to a second round of induction of SpCas9 targeting (Fig. 3a). In the second round 100% of CRT0 and CRT12 strains were insensitive to SpCas9 killing, while the other strains were only partially insensitive: CRT6 (82.5%), CRT11 (73.5%) and CRT13 (58.7%) (Supp. Figure s3). These results indicate that these strains contained heterogeneous populations of which some had a genotypic adaptation that helped them escape SpCas9 mediated killing.

To determine the underlying genetic changes that resulted in resistance to SpCas9-induced killing, we sequenced 14 stably resistant mutants from each experiment, as well as an uninduced control. Additionally, we sequenced the genome of our wild type *E. coli* strain MG1655 in order to compare pre-existing mutations across all of the resistant mutants. Illumina sequencing reads were trimmed and mapped to a reference genome of *E. coli* strain MG1655. SNPs, INDELs and structural changes were detected by CLC Genomic Workbench and a custom-made pipeline^37^. We excluded mutations that were present in all our strains as they were likely pre-existing before the experiment started and unlikely to contribute to the resistance phenotype.

Mutations identified in the chromosome were mainly in non-coding regions or in close vicinity to insertion elements or in repetitive sequences (Supp. Figure s4). Also, mutations in the *glp* gene cluster and *crl* were identified. These genes have been linked to transposition events of insertion elements^38^. Due to the localization of the mutations and overlap between mutation profiles of the control strains and the induced strains, we hypothesized that the chromosomal mutations outside the specific gRNA target sites were not involved in escaping CRISPR induced killing.

In order to test this hypothesis and to identify functionally relevant mutations, we introduced an identical plasmid carrying an intact version of SpCas9 encoding a different selection marker to the genome sequenced resistant strains (Fig. 3b). The new strains were subjected to SpCas9-double stranded DNA break, demonstrating in most cases that the mutant strains were sensitive to this second SpCas9 mediated killing (Fig. 3c), consistent with the hypothesis that most mutations identified in the genome did not contribute to the resistance.

We found that four lineages carrying CRT6 as gRNA were still resistant to killing even after reintroducing the plasmid. These lineages carried chromosomal mutations in the target site of the gRNA providing a plausible explanation for their continued resistance.

The majority of resistant strains we sequenced carried mutations within the plasmid-encoded SpCas9 gene (Fig. 3d). Out of 56 sequenced strains, excluding the control, 46 carried at least one mutation in cas9 (82.14%) and 10 strains harbored even multiple mutations in SpCas9. 29 of all sequenced strains carried genetic alterations in the origin of replication of the plasmid encoding SpCas9. In addition, we also observed six mutations in the theophylline riboswitch that controls the expression of SpCas9. No mutations were identified on the plasmid carrying the gRNA.

Interestingly, we identified only 4 single nucleotide polymorphisms (SNPs) (4.16%) in all our isolates on the plasmids or at the genomic target site. Many genomic changes in these regions (40.62%) stemmed from insertion elements that can be found in the genome of *E. coli* MG1655. These elements only encode the machinery that allows their genomic relocation^39^ and they have previously been observed to play a crucial role in the adaptation process towards multiple environmental stressors including antibiotics^37^ or high production metabolic burden^40,41^. While basic mutation rates are comparable or higher compared to transposition events^42,43^, it has been shown that transposition rates are elevated upon environmental stress and DNA uptake^39,44^. In addition to insertions, 11.46% of the observed mutations were small indels on the plasmids or deletions in the genomic target site, spanning from 783 to 10,565 bps.

Single point mutations have been reported in *cas9* that decrease the DNA cleavage activity of SpCas9^45–47^. In our study the mutated sites that we identified (positions 1571, 2531 and 2735) resulted in early stop codons: Q402X, E722X and E790X. These shortened versions of SpCas9 have not been described before but are likely to impair the overall activity of SpCas as a way to protect the cells from dying.

Notably, we also observed a mutation in the genomic target site of our control strain lacking a PAM suggesting a certain flexibility in the PAM sequence to recognize the genomic targets^48^. Moreover, we also identified mutations in *cas9* in one of our uninduced control strains suggesting that there might be some pre-existing mutations or that the system might be slightly leaky which could result in sufficient selection pressure for genetic adaptations.

In summary, our findings highlight that most spontaneous mutations that rapidly confer resistance to CRISPR-induced killing are insertions and deletions in the cas9 gene as well as its regulatory elements and that multiple target sites of the gRNA prevent the selection of resistance-conferring chromosomal mutations.

## Discussion

CRISPR-based antimicrobials have several challenges to overcome from the delivery of DNA vectors to the intrinsic resistance of cells to repair double stranded breaks. Strategies to understand how to improve the activity of SpCas9 have been explored elsewhere, for example screening libraries of gRNAs to identify chromosomal “hot-spots”^30,31^, engineered versions of SpCas9^49–51^ or inhibition of DNA repair pathways^29^. However, little is known about the mechanism of resistances or basic design rules to improve SpCas9-mediated death in the context of CRISPR-Cas antimicrobials. In this study we focused on circuit design principles and the mutations arising on cells escaping SpCas9-mediated double stranded breaks.

In order to identify a strategy to improve the killing efficiency and reduce the number of escaper cells we tested different gRNAs. We observed there was no correlation between the type of target (coding vs non-coding regions, essential vs. non-essential genes) or the distance from the origin of replication (Fig. 1). This is in line with observations found in other studies with a larger number of gRNA tested^30^. Interestingly, comparison of the predicted activity of the gRNAs tested in this study against the larger dataset used by Guo et al. (2018) indicate that there should be a difference in killing efficiency in the targets based on the chromosomal location (Supp. Figure s1). However, this effect was not observed in the experimental data obtained in this study (Fig. 1b). Additionally, in this study we also focused on evaluating the effect of single vs. multiple target sites. Although the number of target sites did not improve killing of the bacteria (Fig. 1b), we observed that the single-target gRNA (CRT6) allowed mutations in the target site, in contrast to multi-target gRNAs (CRT11, 12 and 13) (Fig. 3). In most cases the majority of the mutations in the non-susceptible strains were accumulated in the plasmid encoding SpCas9, with mutations also arising in the origin of replication and the riboswitch controlling the expression of SpCas9.

We find that lower SpCas9 expression contributes to the transient non-lethal resistance state. We determined that in order to maximize the effect of the gRNA targeting the chromosome it is required to have a specific level of SpCas9 expression (Fig. 2b). Previous reports have demonstrated that overexpression of SpCas9 comes with fitness costs that results in cell toxicity^52–54^; however, we did not observe a significant difference between highly expressed SpCas9 and the viability of the cells (Fig. 2b). It should be taken into account that the level of expression encoded in a vector may be different depending on the target bacteria, which could explain the apparent low efficiency of SpCas9 observed in some organisms^35,55^.

Interestingly, mutations also arise in the control gRNA (CRT0) which was designed to target a region in the chromosome that did not contain the canonical PAM recognized by SpCas9 (NGG). However, it has been previously reported that Cas effectors tend to have more permissive PAMs than previously believed^48^. Therefore, it could be the case that there is some degree of chromosomal damage as a result of imperfect SpCas9 targeting, that results in cell stress. This indicates that it could be desired to use Cas effectors that have been engineered to have stricter PAM recognition and lower off-target effects^49–51^.

Under the conditions used in our study, SpCas9 was the preferred target for mutations in non-susceptible strains (Fig. 3). Re-introduction of an intact CRISPR antimicrobial re-sensitized escaper cells except in a few cases where the gRNA had only a single genomic target (Fig. 3). We can envision the mechanisms of resistance described in this manuscript arising after several cycles or years of treatment using CRISPR-Cas antimicrobials. However, our data suggest that including gRNA to target multiple sites along with orthogonal Cas systems would be a good strategy to reduce mutations.

Finally, it should be mentioned that other mechanisms of resistance could also be linked to the delivery system of CRISPR-Cas antimicrobials (e.g. phage particles)^56^, active mechanisms of bacterial defense (e.g. RM-system)^57^ or the emergence of anti-CRISPR resistance genes^58^. In fact, multiple studies have identified several of these genes located in mobile elements against multiple CRISPR-Cas systems^27,59^.

It is clear that developing antimicrobial drugs is a great challenge and evolution will always work against the efficacy of these treatments. CRISPR-Cas antimicrobials are still being developed, but it is a promising therapy with a potential to impact the way we treat bacterial-derived diseases. Perhaps one day the antibiotics we use for treatment of host–microbial interactions will be considered in a similar manner as chemotherapy in cancer treatment, in the sense that it kills the undesired cells at the cost of damaging healthy cells with unintended side effects.

## Methods

### Cell growth conditions

For most of the experiments overnight cultures were used. The culture was directly inoculated from a glycerol stock in 3 mL LB Medium including antibiotics when required and incubated at 37 °C, 200 rpm for around 18 h. Glycerol stocks were made of an overnight culture from an isolated colony and mixed with 50% glycerol in ratio 1:1. Cells were stored at − 20 °C for short-term (around 4 weeks) or at − 80 °C for long-term storage.

### Cloning of genetic circuit

Construction of pDual plasmids for the different CRISPR targets (CRTs) containing the arabinose inducible gRNA was done by USER cloning. The primers were designed with the AMUSER web tool^60^ (Supp. Table s1 and s2). For amplification of backbone and DNA fragment Phusion U Polymerase from Thermofisher Scientific was used. The chimeric gRNA under a pBAD inducible system and terminator were synthesized from IDT (5′-CTATAACCAGACCGTTCAGCGTTTTAGAGCTAGAAATAGCAAGTTAAAATAAGGCTAGTCCGTTATCAACTTGAAAAAGTGGCACCGAGTCGGTGCTTTTTTT-3′). The backbone of the plasmid was amplified from pSEVA3610, a plasmid that contains a chloramphenicol resistance gene (*cat*), an arabinose inducible expression system and low copy number origin of replication p15A (~ 10 copies)^61^.

Construction of the pCasesn3 plasmid containing SpCas9 was performed in a single step by USER cloning^60^. The fragment containing SpCas9, and the antibiotic resistance gene *aadA* that confers resistance against Spectinomycin, as well as the origin of replication CloDF13 (20–40 copies) were amplified from DS-SPcas addgene ID48645^62^. The theophylline riboswitch was placed in front of SpCas9 using a long forward primer from IDT (5′-AAGTCTAGCGAACCGCACTTAATACGACTCACTATAGGTACCGGTGATACCAGCATCGTCTTGATGCCCTTGGCAGCACCCTGCTAAGGTAACAACAAGATGATGGATAAGAAATACTCAATAGGCTTAGATATCGGCAC-3′). Additionally, a sigma70 constitutive promoter was also introduced using a reverse primer in order to introduce a different promoter for SpCas9 (5′-ctctagTagctagcactgtacctaggactgagctagccgtcaaGTTAGCTGTGCTCTAGAAGCTAGCAG-3′) All constructed plasmids were confirmed by Sanger sequencing.

### Plasmid transformation

Electroporation was used to transform *E. coli* MG1655 strains. To do so an overnight culture was used to inoculate 10 mL of LB media in a ratio of 1:1000. The cells were grown until they reached the exponential growth phase (OD600 0.4–0.6). Thereafter the samples were incubated 15 min on ice and subsequently centrifuged at 4000*g*. The supernatant was removed. The cell pellet was resuspended in 1.5 mL of ice-cold water and transferred into a 2 mL microcentrifuge tube. The samples were centrifuged for 1 min at 4000*g* at 4 °C. The supernatant was removed and the cell pellet resuspended in 1.5 mL of ice-cold MQ water. This step was repeated three times to minimize the salt concentration. In the last step the cells were resuspended in 80 µL of ice-cold MQ water and kept on ice. The plasmids for transformation were stored on ice and 1 ng/µL was transferred to prechilled 1 mm gap electroporation cuvettes. 20 µL of the cells were gently mixed with the target DNA. Cells were electroporated using 1.8 kV and immediately after the pulse, 500 µL of SOC media were added. The cells were transferred into a 1.5 mL microcentrifuge tube and incubated at 37 °C and 200 rpm for 1 h. After the recovery period 100 μL of the cells were plated on LB-agar with the corresponding antibiotics.

### Survival-assay

To investigate the killing efficiency of different gRNAs and SpCas9s a qualitative assay was developed. Overnight cultures of the strains were prepared and diluted up to 10 times in a eight-step dilution series. 5 µL of the dilution series were spotted on agar plates containing the required antibiotics and the recommended inducers (2 mM theophylline and 1% arabinose). As control the same dilution series were spotted on agar plates only containing the required antibiotics. The plates were incubated overnight at 37 °C. For evaluation the colony-forming units (CFU) were counted and normalized with the control data to calculate the survival rate. Technical replicates were made of all experiments and also reproduced three times on independent days.

### Functional-testing of Cas9-mutations

In order to test mutations on a functional level, a new SpCas9 plasmid with a different antibiotic marker for kanamycin was introduced to the escaped mutants by electroporation. Colonies of the transformation were randomly picked and an overnight culture was prepared. The overnight cultures were used to perform the killing assays. The survival rate of the escape mutants with reintroduced SpCas9 was compared to the according untreated origin strain as control. A significant increase of survival rate suggests a mutation independent of SpCas9 sequence.

### Statistical analysis

For statistical analysis (e.g. *t* test) GraphpadPrism8 software was used.

### Computational prediction of gRNA activity

The activity of the gRNA for each CRT was predicted using the model developed by Guo et al. 2018 (accessed on November 2019, https://github.com/zhangchonglab/sgRNA-cleavage-activity-prediction), using the CRISPR targets in Supp. Table s2 and applying default parameters.

### Correlation of CRISPR-Cas predicted killing activity with locations in the chromosome

To see if gRNA target killing efficiency was linked to the number of cutting sites, we used data generated from the genome-wide study by Guo et al. 2018. For each of the 52,563 CRISPRi targets, the number of cutting sites was identified on the *Escherichia coli* str. K-12 substr. MG1655 genome (NCBI Genbank ID U00096.3). This was done using SeqKit v0.13.2^63^. Targets that do not overlap with any coding region (CDS) were deemed to be intergenic. The EcoCyc database^64^ was used to assign gene essentiality, using no observed growth in LB medium after gene knockout as criteria.

### Whole-genome sequencing

Isolated colonies that survived the SpCas9 induced killing were grown in 3 mL LB-broth at 37 °C and 200 rpm overnight. The cultures were centrifuged at 4000*g* for 10 min and the supernatant was discarded. The cells were covered with DNA-shielding buffer (ZYMO research) and sent to BaseClear B.V. for DNA extraction and whole-genome sequencing. Genomic DNA was extracted with a kit from ZYMO research according to the manufacturer's instructions. The genomic library was prepared with a Nextera XT kit from Illumina and 125 paired-end sequencing was performed using Illumina HiSeq 2500. We obtained the resulting fasta reads and subjected them in the following workflow. All sequencing data was deposited in SRA database (accession number: PRJNA706778).

### Sequencing-analysis workflow

First single nucleotide variants (SNPs) as well as small insertions and deletions (INDELs) were identified through CLC Genomics workbench by trimming the reads to ensure high read quality and subsequent sequence alignment to a sequence list containing the *E. coli* U00096 reference genome and the plasmid sequences. SNPs and INDELs were called and only positions with a phred score of 30 or higher at the position of the SNP and three neighboring positions were included. In addition, the frequency of detection had to be at least 80%. Further, we identified large INDELs through the CLC Genomics workbench INDEL function at default settings. The INDELS were considered for the analysis when they occurred with a frequency of at least 80% and in more than 5 reads. In addition, we also used an in-house pipeline to detect large INDELs as described before^37^. Briefly, all open reading frames from the reference genome and plasmids were used to cluster ORFs with cd_hit^65^. The cluster cut-off was set to 90% identity and coverage. FASTX_toolkit was used to quality filter the fasta reads from this study and reads with a minimum quality of 30 were blasted against the clustered ORFs. Reads with 90% coverage that mapped to two clusters with an overlap of between 10 and 90% were kept for further analysis. INDELs were only counted when the reads that mapped to two clustered ORFs were not adjacent and when they were observed in at least 5 reads. INDELs that were detected by multiple parallel analysis were only counted once. We also identified structural variants such as inversions or regions with multiple breakpoints by CLC Genomic Workbench with default parameters.

## Supplementary Information

Supplementary Information.
