# Supplementary material for: Bacterial resistance to CRISPR-Cas antimicrobials
Source: Sci Rep. 2021 Aug 26;11:17267. doi: 10.1038/s41598-021-96735-4 (PMC8390487; doi:10.1038/s41598-021-96735-4)
Supplement: Supplementary file 1 — Supplementary Information. [file 41598_2021_96735_MOESM1_ESM.pdf]

Bacterial resistance to CRISPR-Cas antimicrobials

Ruben V. Uribe[1,#], Christin Rathmer[1,#], Leonie Johanna Jahn[1,#], Mostafa Mostafa Hashim Ellabaan[1], Simone S. Li[1,2], Morten Otto Alexander Sommer[1*]

[1] Novo Nordisk Foundation Center for Biosustainability, Technical University of Denmark, Kgs. Lyngby, Denmark
[2] School of Chemistry and Molecular Biosciences, University of Queensland, Queensland, Australia

# These authors contributed equally to this work.

*Address correspondence to Morten O.A.Sommer, msom@bio.dtu.dk

SUPPLEMENTARY DATA

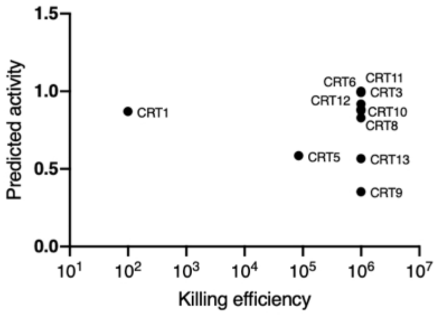

Figure s1. Observed CRISPR-Cas killing activity vs. predicted activity. The results from the killing assays were compared against the predicted values for the different CRISPR targets (CRTs) used in this study (table s2).

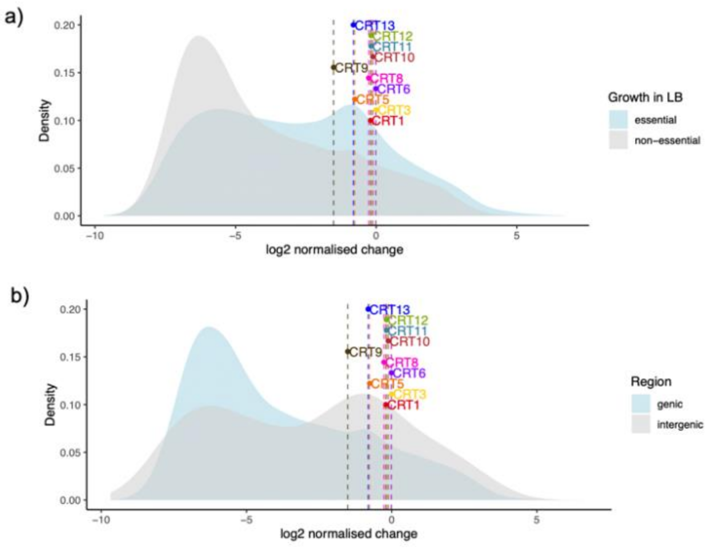

Figure s2. Comparison of CRISPR-Cas killing activity with locations in the chromosome using data generated from the genome-wide study by Guo et al. 2018. a) essential vs. non-essential chromosomal location. b) genic (coding) vs. intergenic (non-coding) regions. The EcoCyc database (Keseler et al., 2017) was used to assign gene essentiality. Targets that do not overlap with any coding region (CDS) were deemed to be intergenic.

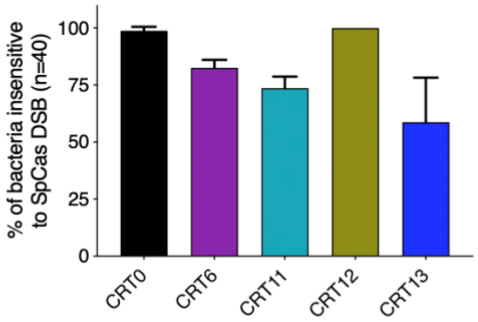

Figure s3. Percentage of bacteria that acquired a mutation inactivating the effect of the antimicrobial. 40 escaper colonies of each experiment from: CRT0 (control), CRT6, CRT11, CRT12 and CRT13 after being exposed to a second round of SpCas9-induced chromosomal break.

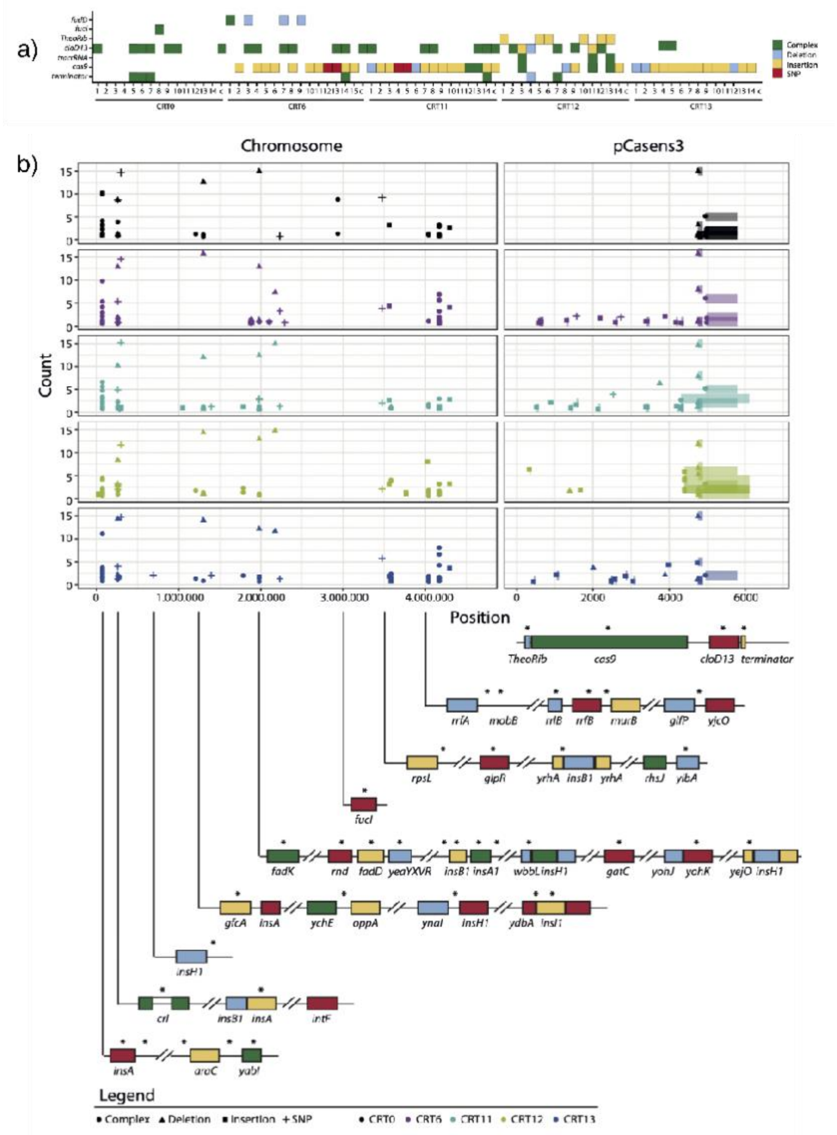

Figure s4. Mutation profile of escaper strains. a) Potentially resistance causative mutations on the plasmid (terminator, cas9, tracrRNA, origin of replication cloD13, theophyline riboswitch TheoRib) and in the target region of the chromosome (CRT0 and fucI; CRT6 and fadD) for 14 escapers and one control for CRT0 (control), CRT6, CRT11, CRT12 and CRT13. The colors highlight different mutation types: SNPs are visualised in red; Insertions are represented in yellow, deletions are visualized in blue and complex rearrangements are depicted in green. b) Overview of the entire mutational landscape across the genome and plasmid (pCasens3) of CRT0 (black), CRT6 (purple), CRT11 (blue), CRT12 (green) and CRT13 (dark blue). Number of mutations in one locations were counted across the 14 replicates and the control. Mutational hotspots were further investigated: the genomic regions are diplayed in more detail and mutations are highlighted by stars. Most chromosomal mutations were located in noncoding regions, close to insertion elements, in repetitive regions or in regions with homology to the plasmid - suggesting that they might be read mapping artefacts.

Table s1. Strains and plasmids

Strain	Description	Reference
Escherichia coli Top 10	F- *mcr*A Δ(*mrr-hsd*RMS-mcrBC) Φ80l*ac*ZΔM15 Δ *lac*X74 *rec*A1 *ara*D139 Δ(*araleu*)7697 *gal*U *gal*K *rps*L (Str^R) *en*dA1 *nup*G	Thermofisher Scientific®
Escherichia coli MG1655	Wild-type *E. coli* MG1655 derived from *E. coli* K12	Blattner et. al. 1997

Plasmids	Description	Reference
pCaSens3Spec	Spec^r ;bacterial *cas9*; RBS_{Theo}; CloDF1	This study
pCaSens3Kan	Kan^r ;bacterial *cas9*; RBS_{Theo}; CloDF1	This study
PCaSens6Spec	Spec^r ;bacterial *cas9*; yeast-like *cas9*; RBS_{Theo}; CloDF1	This study
pCaSens7Spec	Spec^r ;yeast-like *cas9*; RBS_{Theo}; CloDF1	This study
pDual4CRT#0	Cm^r; gRNA (5'agttctggcaagcgcggtaa 3') P_{BAD}; p15a	This study
pDual4CRT#1	Cm^r; gRNA (5'tggggctatcgataaactcg 3') P_{BAD}; p15a	This study
pDual4CRT#3	Cm^r; gRNA (5'cacttcagttctttctcatc 3') P_{BAD}; p15a	This study
pDual4CRT#5	Cm^r; gRNA (5'ctacgcgctggataccttca 3') P_{BAD}; p15a	This study
pDual4CRT#6	Cm^r; gRNA (5' tgacgactgacttaacgctc 3') P_{BAD}; p15a	This study
pDual4CRT#8	Cm^r; gRNA (5' cgcggatcaacctgttcttc 3') P_{BAD} ; p15a	This study
pDual4CRT#9	Cm^r; gRNA (5'cgaactcgcgaccccgacct 3') P_{BAD}; p15a	This study
pDual4CRT#10	Cm^r; gRNA (5' tttgtaggcctgataagacg 3') P_{BAD}; p15a	This study
pDual4CRT#11	Cm^r; gRNA (5' gacgcggcaagcgtcgcatc 3') P_{BAD}; p15a	This study
pDual4CRT#12	Cm^r; gRNA (5' aagacgcgccagcgtcgcatc 3') P_{BAD}; p15a	This study
pDual4CRT#13	Cm^r gRNA(5' agacgcgccagcgtcgcatc 3') P_{BAD}; p15a	This study

Table s2. Properties of CRTs used in this study.

CRT	# Cutting Sites	Sequence	PAM	Coding vs. Non-coding	Essentiality[1]	Killing efficiency (%)	Predicted gRNA efficiency [2]	
							Total	Scored[3]
1	1	tggggctatcgataaactcg	agg	C	Non-essential	99.9995	27.2473665	0.87038401
3	1	cacttcagttctttctcatc	ggg	N	Non-essential	99.9958	28.6472928	1
5	1	ctacgcgctggataccttca	cgg	C	Non-essential	84.963	24.1713112	0.58557906
6	1	tgacgactgacttaacgctc	agg	C	Non-essential	99.9999	28.5569499	0.99163535
8	2	cgcggatcaacctgttcttc	tgg	C	Non-essential	99.9984	26.8053636	0.82945997
9	4	cgaactcgcgaccccgacct	tgg	C	Essential	99.9973	21.661976	0.35324543
10	5	tttgtaggcctgataagacg	cgg	N	Non-essential	99.9992	27.7691709	0,.1869669
11	10	gacgcggcaagcgtcgcatc	agg	N	Non-essential	99.9961	27.4042331	0.88490793
12	25	aagacgcgccagcgtcgcatc	agg	N	Non-essential	99.9996	27.3034114	0.87557309
13	25	taggtgaagtccctcgcgga	tgg	C	Essential	99.9961	23.9639611	0.56638099

[1]Essentiality of a gene was based on the information available on Ecocyc.

[2]The predicted efficiency of the CRTs was determined using the model developed by Guo et al. 2018

[3]and was scored against the range of values obtained for this dataset.
